# Conductive resins improve charging and resolution of acquired images in electron microscopic volume imaging

**DOI:** 10.1038/srep23721

**Published:** 2016-03-29

**Authors:** Huy Bang Nguyen, Truc Quynh Thai, Sei Saitoh, Bao Wu, Yurika Saitoh, Satoshi Shimo, Hiroshi Fujitani, Hirohide Otobe, Nobuhiko Ohno

**Affiliations:** 1 Department of Anatomy and Molecular Histology, Interdisciplinary Graduate School of Medicine and Engineering, University of Yamanashi, Chuo-city, Yamanashi 409-3898, Japan; 2 Department of Occupational Therapy, Health Science University, Fujikawaguchiko, Yamanashi 401-0380, Japan; 3 Gatan Japan, Koto-ku, Tokyo 135-0033, Japan; 4 Asahi Kasei Chemicals Corporation, Kawasaki-city, Kanagawa 210-0863 Japan; 5 Center for Multidisciplinary Brain Research, National Institute for Physiological Sciences, Okazaki, Aichi 444-8787, Japan

## Abstract

Recent advances in serial block-face imaging using scanning electron microscopy (SEM) have enabled the rapid and efficient acquisition of 3-dimensional (3D) ultrastructural information from a large volume of biological specimens including brain tissues. However, volume imaging under SEM is often hampered by sample charging, and typically requires specific sample preparation to reduce charging and increase image contrast. In the present study, we introduced carbon-based conductive resins for 3D analyses of subcellular ultrastructures, using serial block-face SEM (SBF-SEM) to image samples. Conductive resins were produced by adding the carbon black filler, Ketjen black, to resins commonly used for electron microscopic observations of biological specimens. Carbon black mostly localized around tissues and did not penetrate cells, whereas the conductive resins significantly reduced the charging of samples during SBF-SEM imaging. When serial images were acquired, embedding into the conductive resins improved the resolution of images by facilitating the successful cutting of samples in SBF-SEM. These results suggest that improving the conductivities of resins with a carbon black filler is a simple and useful option for reducing charging and enhancing the resolution of images obtained for volume imaging with SEM.

Electron microscopy has been an indispensable approach for acquiring ultrastructural information in life science. Recent advances in volume imaging with scanning electron microscopy (SEM) have enabled the imaging of an unprecedentedly large volume of biological samples and also the rapid acquisition of 3D structural information of the nervous system^1^,^2^. Multiple approaches including serial block-face SEM (SBF-SEM), focused ion beam SEM, array tomography, and an automated tape-collecting ultramicrotome are available and used in growing numbers of studies for the volume imaging. These methods are powerful tools in neuroscience imaging brain tissues to achieve wiring map of brain circuitry^3^,^4^,^5^,^6^,^7^, and also provided ultrastructural information of cells and organelles in various tissues^8^,^9^,^10^.

Most biological samples are non-conductive in nature; therefore, increasing the conductivity of samples is critical for SEM observations in order to avoid the charging of samples. Therefore, prior to volume imaging under SEM, intensive osmium reactions and *en bloc* staining with heavy metals such as uranium and lead are used in tissue preparation before embedding to avoid severe charging and achieve high contrast in serial image acquisition^11^,^12^,^13^. Recent effort towards homogenous staining of bulk tissues enabled acquisition of images from the brain tissues for reconstruction of very large volume^14^,^15^. However, even with these approaches, imaging at higher magnifications and longer dwelling times for a better signal-to-noise ratio need higher electron doses and may be hampered by sample charging and damage, which are particularly prominent in SBF-SEM imaging^16^,^17^,^18^. It currently remains unclear whether increasing the conductivities of resins, which are typically non-conductive, reduces charging and sample damage and is beneficial for successful volume imaging with SEM.

In the present study, we introduced carbon-based conductive resins for volume imaging of biological specimens with SBF-SEM. Conductive resins were produced by adding a carbon black filler to standard resins used for electron microscopic observations of biological specimens. When these conductive resins were used in imaging small mouse tissues, carbon black mostly localized at the periphery of the tissues and infiltrated some of the vessels/tubules. However, embedding in conductive resins significantly improved the charging of samples. It also facilitated the successful cutting of samples and enhanced the resolution of the images obtained. These results suggest that conductive resins are a simple option for electron microscopic imaging of samples prone to charging, and may support the wider application of volume imaging with SEM.

## Results

**Figure 1:**
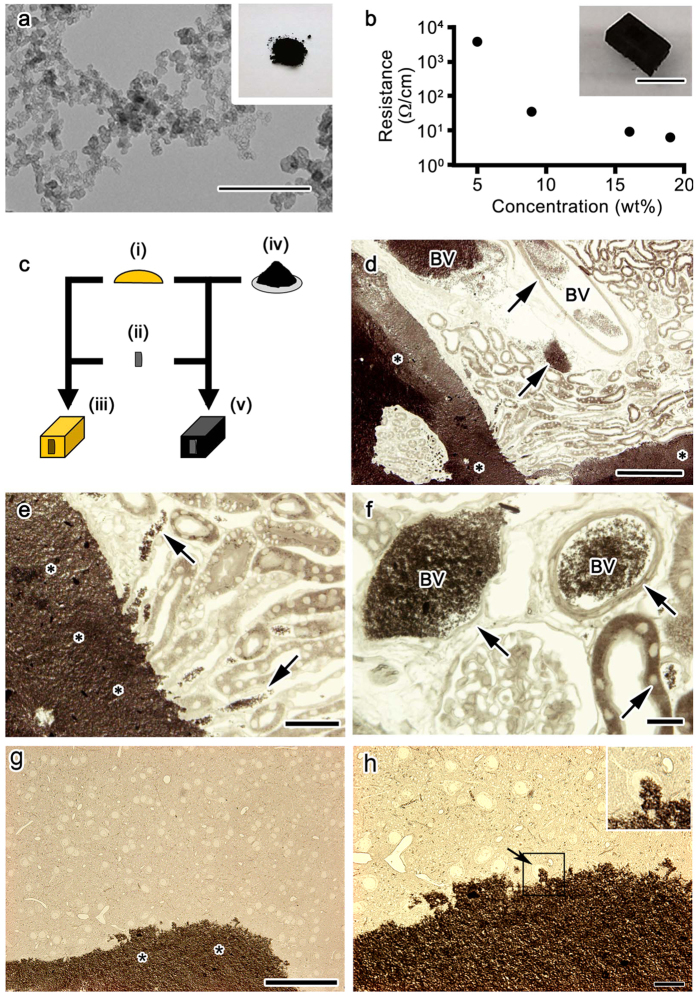
Conductive resins were produced by the addition of a carbon filler. Ketjen black appeared as large agglomerates in a transmission electron microscopic image (**a**), and its powder (**a**, inset) was added to standard resins used for biological specimens in order to produce conductive resin blocks (**b**, inset). A scatter plot shows that the resistance of the block was decreased as the filler concentration increased (**b**). In order to address the effects of carbon black, control resin was first prepared (**c**, i), and *en bloc* stained samples (**c**, ii) were embedded in either the control resin (**c**, iii) or conductive resins (**c**, v) prepared by adding Ketjen black (**c**, iv) to the control resin. Light microscopic images of mouse kidney (**d–f**) and brain (**g,h**) samples embedded in conductive resins showed that carbon black, which appeared as dark granular aggregates, was largely distributed outside the tissues (**d,e,g**, asterisks), but was also found in large and small vessels (**d–f**,**h**, arrows). The area marked with a rectangle is enlarged in the inset (**h**). BV: blood vessels. Bars: 500 nm (**a**), 500 μm (**b**), 200 μm (**d**), 100 μm (**g**), 50 μm (**e**), or 20 μm (**f,h**).

Increases in the conductivities of the resins were assumed to decrease charging and enhance the quality of the images obtained. We initially attempted to develop electron-conductive resins by mixing resins and several types of ionic liquids, but were unable to achieve a sufficient decrease in resin resistance (data not shown). Therefore, we focused on the carbon black conductive filler, Ketjen black, because it is commonly used and increases the conductivity of materials. Ketjen black macroscopically appeared as a black powder (Fig. 1a, inset), and was observed as fibrous agglomerates of carbon under TEM (Fig. 1a). When it was added to Quetol 812 or Plain resin, the cured resins appeared as dark blocks (Fig. 1b, inset). Although the surface resistance of cured conductive Plain resin inversely decreased when more carbon was added (Fig. 1b), the viscosity of the uncured resins increased in proportion to the amount of Ketjen black added to the resin (not shown), and we subsequently selected conductive resins containing approximately 7% and 17–19% (w/v) of carbon for Quetol 812 and Plain resin, respectively. In order to determine the effects of the carbon filler on serial block-face imaging under SBF-SEM, sets of various tissues were stained at the same time, embedded in either control resin or conductive resins (a mixture of control resin and Ketjen black), and observed under various conditions (Fig. 1c). We included brain and kidney tissues which have more resin-only spaces and prone to charging problems^19^. Light microscopic pictures of 1-μm-thick sections obtained from the samples infiltrated with normal Plain resin and embedded in the 17–19% conductive Plain resin revealed that the dark agglomerates of carbon were clearly observed at the light microscopic level, and were mostly localized around the periphery of tissues (Fig. 1d,e). Some agglomerates occasionally infiltrated into the large and small vessels of the samples (Fig. 1d–f); however, the dark carbon was not observed inside the cells. Occasional penetration into vessels and little penetration into cells of the carbon were common in the brain tissues (Fig. 1g,h).

**Figure 2:**
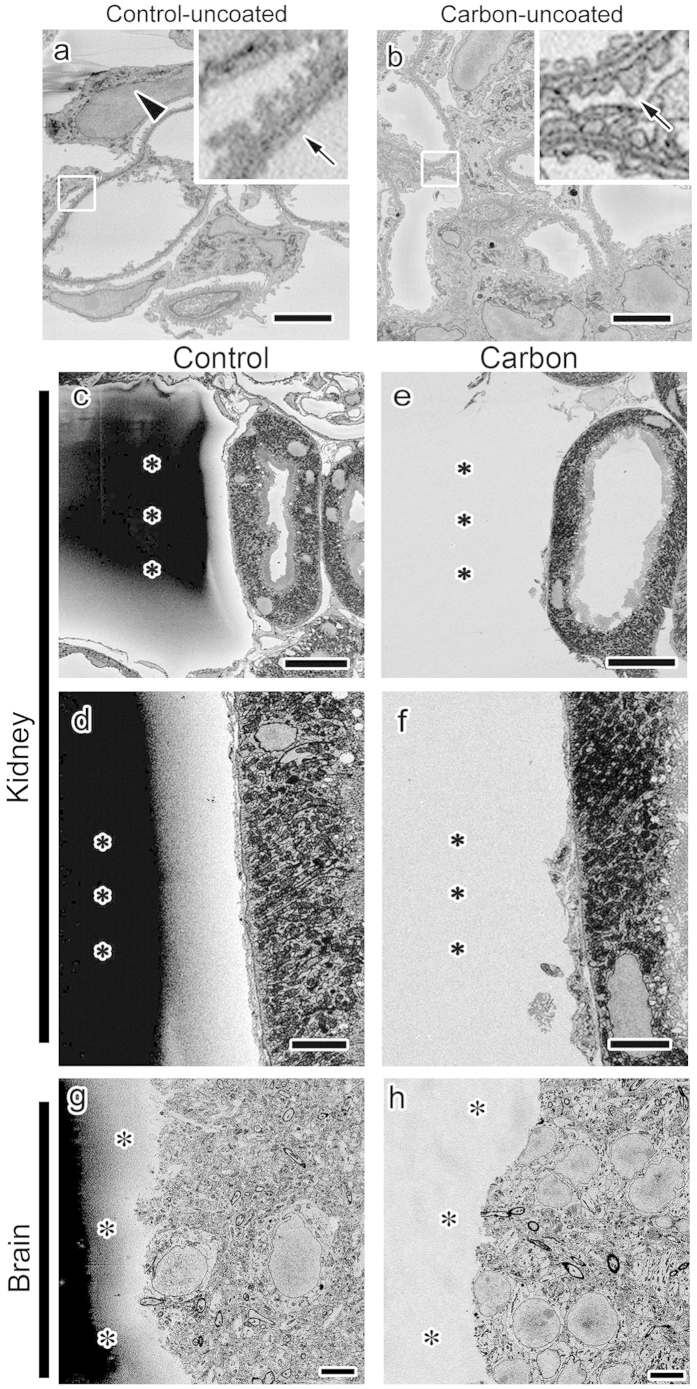
Conductive resins reduced the charging of block-face observations. Scanning electron microscopic block-face images of mouse kidney and brain tissues embedded in Quetol 812 (**a,b,g,h**) or Plain resin (**c–f**) with (**b,e,f,h**) or without (**a,c,d,g**) carbon and observed with (**c–h**) or without (**a,b**) the conductive surface treatment of gold sputtering showed that, without gold sputtering, images of samples embedded in Quetol 812 (Control-uncoated) were distorted (**a**, arrowhead) and blurred (**a**, arrow) due to charging, whereas these issues were less severe (**b**, arrow) in samples embedded in conductive resins (Carbon-uncoated). Furthermore, charging in the resin-only areas (**c,d,g**, asterisks), which is obvious in Plain resin or Quetol 812 (Control), was significantly reduced when conductive Plain resin or Quetol 812 (Carbon) was used (**e,f,h**, asterisks). Bars: 2 μm (**a,b**), 20 μm (**c,e**), 10μm (**g,h**) or 5 μm (**d,f**).

Although carbon mostly localized around the tissues, embedding in conductive resins may have affected images inside the tissues. When the block face of mouse kidney samples embedded in Quetol 812 was imaged without the surface conductive treatment of gold sputtering, severe charging blurred and deformed images of the glomerular capillary walls (Fig. 2a). In contrast, such deformation and blurring were diminished in those embedded in the 7% conductive Quetol 812 under the imaging condition at a higher resolution (Fig. 2b). Furthermore, when the kidney and brain samples were imaged with gold sputtering, the charging of areas without tissues was prominent in Plain resin (Fig. 2c,d) and Quetol 812 (Fig. 2g) without carbon black, but minimal in 17–19% conductive Plain resin (Fig. 2e,f) and 7% conductive Quetol 812 (Fig. 2h). These results demonstrated that the conductive resins produced by the addition of the carbon black filler significantly reduced the charging of block-face imaging.

**Figure 3:**
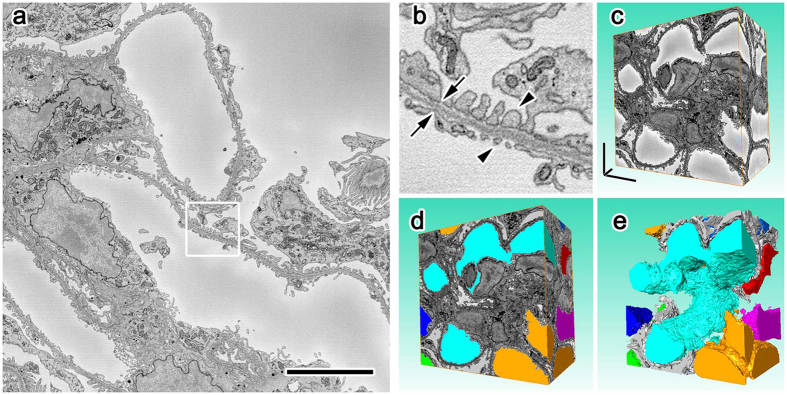
Serial imaging and 3D reconstruction using the carbon-based conductive resin and SBF-SEM. An image at low (**a**) and high (**b**) magnifications and 3 dimensional reconstructions of glomerular capillaries (**d,e**, various colors) and Bowman’s space (**d,e**, gray color) obtained from the mouse kidney embedded in conductive resins clearly showed cellular processes (**b**, arrowheads) and basement membranes (**b**, arrows). Bars: 5 μm.

**Figure 4:**
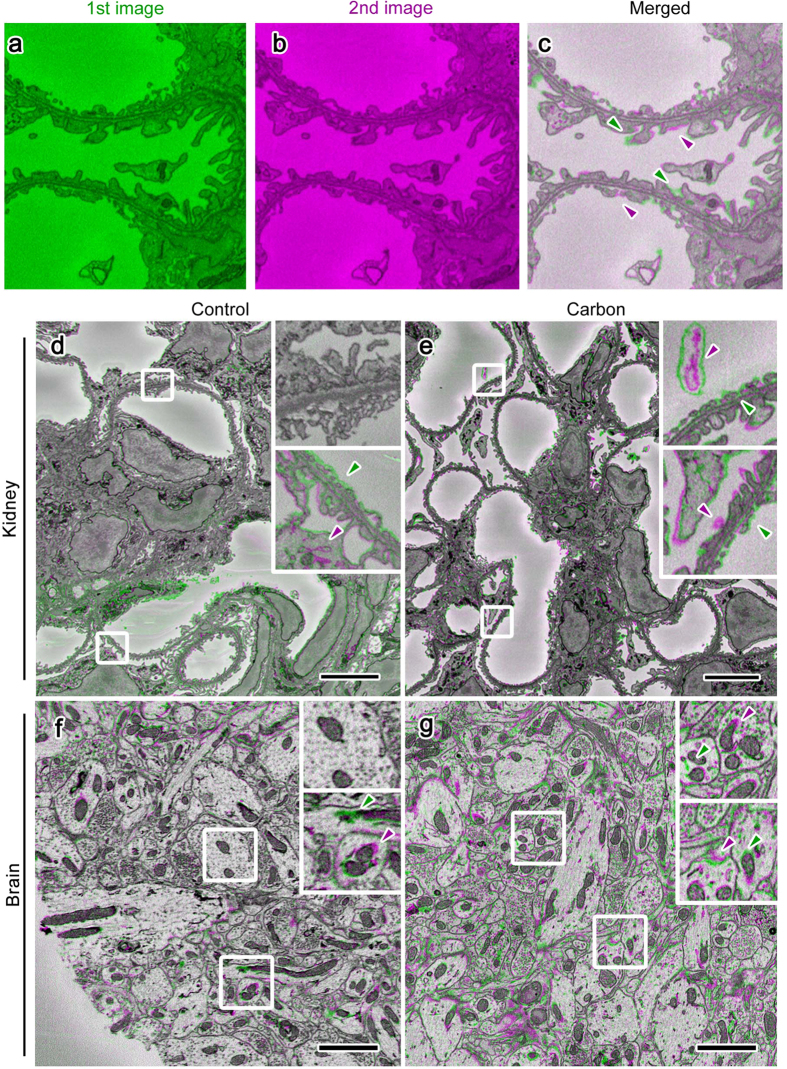
Conductive resins facilitated successful serial sectioning in SBF-SEM. Merging of the first frame, colored green (**a**), and the second frame, colored magenta (**b**), showed that appeared and disappeared profiles in a set of consecutive images were colored green (**c**, green arrowheads) and magenta (**c**, magenta arrowheads), respectively. Merged images obtained from mouse kidney (**d,e**) and brain (**f,g**) tissues embedded in Plain resin (**d,e**) or Quetol 812 (**f,g**) with (Carbon; **e**,**g**) or without (Control; **d**,**f**) carbon black and observed under the same conditions showed that sectioning was heterogeneous in Control resin (**d**,**f**, green and magenta arrowheads), in which colors only appeared in the lower part, but was homogenous in Carbon (**e**,**g**, green and magenta arrowheads). Bars: 5 μm (**d,e**) or 2 μm (**f,g**).

**Figure 5:**
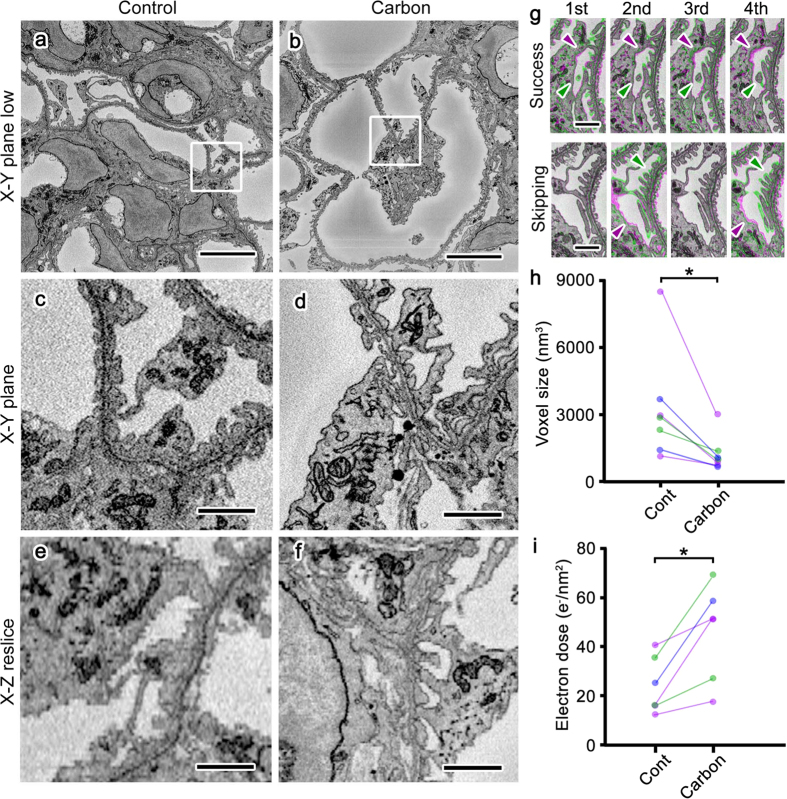
Conductive resins achieved imaging at a higher resolution. Serial block-face images obtained at the highest possible resolution from mouse kidney samples in Control (**a,c,e**) or Carbon (**b,d,f**) showed that resolution was higher in Carbon than in Control, and this difference was more prominent when X-Z resliced images were compared (**e,f**). In colored serial images, as in Fig. 4d–g, the appearance and disappearance of profiles in consecutive cuttings (1st–4th) were observed in successful serial imaging (Success; **g**, upper panels, green and magenta arrowheads), whereas this appearance and disappearance happened in every other cutting in unsuccessful serial imaging (Skipping; **g**, lower panels, green and magenta arrowheads). The smallest possible voxel sizes (**h**) and the highest electron doses (**i**) in the successful serial imaging of kidney (**h**,**i**, blue dots and lines) and brain tissues (**h**,**i**, purple dot and line) fixed with transcardial perfusion and kidney tissues fixed with resection and immersion (**h**,**i**, green dots and lines) showed that the voxel sizes and the electron doses are smaller (the resolution is higher) and higher (more electron can be irradiated in the successful serial imaging), respectively, in all tissues when they were embedded in Carbon than in Control. *p < 0.05 in Wilcoxon matched-pairs signed rank test. N = 7 (**h**) or 6 (**i**). Each set of tissues was prepared at the same time and observed under the same conditions but different XY-resolutions and slicing thickness (**h**) or XY-resolution and dwelling time (**i**). Bars: 10 μm (**a,b**), 1 μm (**c–f**), or 2 μm (**g**).

In order to determine whether less charging by conductive resins benefited serial image acquisition, we obtained SBF-SEM images under the same observation conditions from sets of samples prepared at the same time and embedded in resin with (conductive) or without (control) carbon black. Serial images of tissues including mouse kidneys at a subcellular resolution were successfully obtained in SBF-SEM (Fig. 3a–e). When the serial images obtained were colored and merged to evaluate success in consecutive cutting by the colors of the profiles (Fig. 4a–c), the kidney samples in 17–19% conductive Plain resin and the brain samples in 7% conductive Quetol 812 were cut smoothly and uniformly without detectable skipping, even under the observation conditions with intensive electron beam irradiation in which samples in control resin could not be cut uniformly (Fig. 4d–g). In addition, the smallest possible voxel sizes for successful serial imaging were smaller in samples embedded in 17–19% conductive resin (Fig. 5b,d,f) than in control resin (Fig. 5a,c,e). The difference in resolution was apparent when serial sections were resliced to show X-Z plane images (Fig. 5e,f), and the morphologies of cells and organelles were clearer in samples embedded in conductive resins (Fig. 5f). When successful serial imaging was defined as smooth cutting without skipping (Fig. 5g) or partial cutting (Fig. 4d,f) in 5 consecutive images, the better resolution observed in conductive resins was common among tissue samples that included mouse kidneys and brains and were prepared at the same time, embedded in either control or conductive resins, and imaged under the same accelerating voltages, probe current, and dwelling time (Fig. 5h). Furthermore, the successful serial imaging was possible even under higher electron doses in samples embedded in conductive resins, when each set of tissue samples were imaged under the same conditions but different resolution and dwelling time (Fig. 5i). Taken together, these results demonstrated that carbon-based conductive resins improved charging and image resolution in volume imaging using SBF-SEM.

## Discussion

In the present study, conductive resins were developed by adding a carbon black filler to resins commonly used in electron microscopic observations of biological specimens. The conductive resins were applied to the acquisition of serial images obtained with SBF-SEM. Embedding in conductive resins significantly reduced the charging of tissue blocks during imaging and also enhanced the resolution of the images obtained. These results indicated that increasing the conductivities of the resins with carbon black filler provided a simple and beneficial option for improving the image quality of volume imaging through less charging and higher resolution.

Conductive resins improved the charging of the inside and outside of tissues in SBF-SEM. SBF-SEM imaging requires thorough cutting of the block face with diamond knives and, thus, is relatively prone to charging when the cut surface is largely non-conductive^19^,^20^. A conductive surface coating between each cut was previously shown to reduce charging in SBF-SEM imaging^21^, but needs specialized equipment and takes additional time for image acquisition. The usage of conductive resins represents another solution for reducing charging, and may be produced simply by the addition of carbon black fillers to standard resins. Since some resins are relatively resistant to electron damage, it has yet to be determined which are preferred for the imaging of certain tissues with a carbon black filler^22^. In addition, we have tried Ketjen black in Durcupan, but the carbon could not be homogenously dispersed in Durcupan as in Quetol 812 and Plain resin (data not shown). Optimal methods to mix the Ketjen black may vary among different types of resins. Furthermore, future studies are needed in order to examine whether other approaches, such as surface coating and beam deceleration^21^,^23^, are beneficial in combination with conductive resins.

The results of the present study suggested that the application of a conductive resin to serial block-face observations under SEM improved the resolution of the images acquired. It currently remains unknown why the usage of conductive resins had favorable effects on images at higher resolutions, and multiple mechanisms have been suggested. One possibility is less damage to the samples caused by a higher dose of the electron beam because charging negatively affects the physical properties of resins and samples^24^,^25^. Reduced charging may preserve the hardness of samples and enable thinner sectioning during imaging at higher resolutions. Another possibility is the effects of carbon black fillers on the physical properties of the resins. Previous studies reported that the addition of a carbon black filler to resins and polymers changed the physical properties and conductivities of the materials, and resulted in increased toughness of the materials^26^,^27^. The addition of a carbon black filler may enhance the hardness of standard resins, thereby facilitating thinner sectioning of the samples.

One drawback of carbon-based conductive resins is limited penetration into tissues and cells. We did not observe significant increases in penetration even when the tissues were incubated overnight in 3% conductive resin, which has lower viscosity (data not shown). Therefore, the beneficial effect of carbon could be less obvious if the samples become larger and contain few regions with carbon. Since carbon black infiltrated some vessels in kidney tissues, conductive resins may be more effective for thin, small or porous tissues such as vibratome sections or cultured cells that generally have more resin-only area around cells. The limited penetration of carbon may be largely due to the size and form of the conductive carbon fillers, and also partly to the higher viscosities of uncured conductive resins with a larger quantity of carbon. The conductivities of polymers with carbon black fillers have been shown to depend on the structures as well as concentrations of fillers^28^,^29^,^30^. Ketjen black may form relatively large agglomerates, which may contribute to increases in conductivities at relatively low concentrations in return for poor penetration^31^,^32^. Although other carbon black fillers may require higher concentrations to increase the conductivities of resins, the combination of Ketjen black and smaller carbon black aggregates may improve the penetration and conductivities of specimens and further enhance image quality.

## Materials and Methods

### Reagents for electron microscopic observations

Fixatives including glutaraldehyde and aqueous OsO_4_, Plain resin, and the Quetol 812 kit were purchased from Nisshin EM (Tokyo, Japan). Lead nitrate was obtained from Kanto Chemicals (Tokyo, Japan). Thiocarbohydrazide (TCH) was purchased from Sigma Aldrich (St. Louis, MO, USA). Other reagents were obtained from Nacalai Tesque (Kyoto, Japan).

### Animals

All experimental procedures were approved by the University of Yamanashi Animal Care and Use Committee, and conducted in accordance with the Guidelines for the Care and Use of Experimental Animals by the University of Yamanashi. Three adult DBF-1 (for brain) or two C57BL/6 (for kidney) mice, purchased from Japan SLC (Shizuoka, Japan) and housed under free access to food and water, were transcardially perfused with 4% paraformaldehyde and 0.5% glutaraldehyde in 0.1M phosphate buffer (pH 7.4) under pentobarbital anesthesia. Tissues of the cerebrum and kidney were collected and immersed in the same fixative at 4 °C overnight. Kidney tissues from two C57BL/6 mice were resected without transcardial perfusion, cut into small pieces with a razor blade in ice cold 0.1M phosphate buffer (pH 7.4) with 2.5% glutaraldehyde, and immersed in the same fixative at 4 °C over several nights. The 7 groups of tissues were cut small (<1mm in size), washed with phosphate buffer saline (PBS) or 0.1M cacodylate buffer, and used for specimen preparation.

### Electron microscopic specimen preparation

*En bloc* heavy metal staining was performed as reported previously with some modifications^33^. Briefly, tissues were washed with either PBS or cacodylate buffer (pH 7.4) for 10–15 min with a total of 5 buffer changes. Tissues were then treated with 2% OsO_4_ in 0.15% K_4_[Fe(CN)_6_] for 1 hr on ice, and 0.1% thiocarbohydrazide for 20 min and 2% OsO_4_ for 30 min at room temperature. Thereafter, some tissues were treated with 1% uranyl acetate at 4 °C overnight. Tissues were then treated with lead aspartate solution at 70 °C for 30 min. Each of these treatments was followed by washing 5 times with double distilled water for 10–15 min. Tissues were dehydrated in a graded series of ethanol (60%, 80%, 90% and 95%, 5 min each), infiltrated with acetone dehydrated with a molecular sieve, a 1:1 mixture of resin and acetone, and 100% resin. Resins were prepared from the Quetol 812 kit (Nisshin EM, Tokyo, Japan) by mixing 9.7 ml Quetol 812, 3.3 ml DDSA, 6.6 ml MNA and 0.3 ml DMP-30 for 20 ml, or Plain resin (Nisshin EM, Tokyo, Japan) following the manufacturer’s instructions. The samples in resin were placed in a mold, and cured at 70 °C overnight. Light microscopic pictures were obtained by observations of 1-μm-thick sections under BX-61 (Olympus, Tokyo, Japan).

Regarding conductive resins, Ketjen black powder was manually mixed with Plain resin or Quetol 812 just before use. The Ketjen black powder is freely available by contacting authors, Nobuhiko Ohno or Hirohide Otobe, but commercially available Ketjen black with similar properties could be used as substitution. Following infiltration with normal resin or 3% (w/v) conductive Quetol 812, tissues were embedded in 7% (w/v) conductive Quetol 812 or 17–19% (w/v) conductive Plain resin. TEM images of Ketjen black were obtained by dispersing it in 100% ethanol with sonication, followed by observations with TEM after drying the mixture on copper grids. The surface resistance of the resins was measured using the 4 point probes method.

### Observations with SBF-SEM and data analyses

One or two blocks from each group (each of perfusion-fixed kidneys and brains and immersion-fixed kidneys obtained from different animals and embedded in either normal or conductive resins) were trimmed and mounted on aluminum rivets with conductive glue (CW-2400, Circuitworks). The surfaces of trimmed samples were treated with gold sputtering to increase conductivity, and imaged under various imaging conditions (total 129 sessions of serial image acquisition) in Merlin or Sigma (Carl Zeiss) equipped with 3View (Gatan). Imaging in Merlin was performed under a constant probe current (150pA) and the crossover-free mode. Imaging in Sigma was performed with a 30-μm aperture. The other parameters for imaging conditions in the figures are summarized in Table 1. The serial images obtained were handled with ImageJ and Fiji plugins (http://fiji.sc/wiki/index.php/Fiji), and segmentation and image analyses were performed in TrakEM2^34^ and Amira (FEI Visualization Science Group, Hillsboro, OR, USA). The smallest possible voxel size was determined by (1) imaging the samples to acquire multiple series of images under the same conditions but different pixel resolutions and slice thicknesses, and (2) finding out the smallest voxel sizes (=(pixel resolution)^2^ × slice thickness) where successful serial imaging was possible. The successful serial imaging was defined as smooth cutting without skipping (Fig. 5g) or partial cutting (Fig. 4d,f) in 5 consecutive images. The electron dose of the imaging was calculated from probe current (A), pixel dwelling time (s) and pixel resolutions (nm^2^), as reported previously^16^. The highest electron dose was determined by (1) imaging each set of samples to acquire multiple series of images under the same conditions but different pixel resolutions and dwelling time, and (2) finding out the highest electron dose where successful serial imaging was possible. Statistical comparisons were made by Wilcoxon matched-pairs signed rank test.

## Additional Information

**How to cite this article**: Nguyen, H. B. *et al*. Conductive resins improve charging and resolution of acquired images in electron microscopic volume imaging. *Sci. Rep.* **6**, 23721; doi: 10.1038/srep23721 (2016).

## Figures and Tables

**Table 1:** Summary of imaging conditions.

Panel	Resin	Voltage (kV)	Image size (pixels)	Dwell time (us)	X-Y resolution (nm)	Z-step (nm)
Fig. 2a	Normal	1.5	4096^2^	1	7.5	50
Fig. 2b	Conductive	1.5	4096^2^	1	5.7	40
Fig. 2c–f	Normal Conductive	1.5	4096^2^	3	26	–
Fig. 2g,h	Normal Conductive	1.4	4096^2^	2	25	–
Fig. 3a–e	Conductive	1.4	4096^2^	1	5.7	30
Fig. 4d,e	Normal Conductive	1.5	8192^2^	1	4	40
Fig. 4f,g	Normal Conductive	2	3072^2^	1	4.8	40
Fig. 5a,c,e	Normal	1.4	4096^2^	1	8.6	50
Fig. 5b,d,f	Conductive	1.4	4096^2^	1	5.7	30
Fig. 5h	Normal	1.5	8192^2^	1	5.7	40
	Conductive	1.5	8192^2^	1	4	40
	Normal	1.4	4096^2^	1	8.6	50
	Conductive	1.4	4096^2^	1	5.7	30
	Normal	1.4	4096^2^	1	10.3	80
	Conductive	1.4	4096^2^	1	6.5	70
	Normal	1.4	4096^2^	2	5.9	50
	Conductive	1.4	4096^2^	2	6	40
	Normal	2	3072^2^	1	7.7	40
	Conductive	2	3072^2^	1	5.9	40
	Normal	2	3072^2^	1	7.7	50
	Conductive	2	3072^2^	1	4.8	40
	Normal	2	3072^2^	1	4.8	50
	Conductive	2	3072^2^	1	4.3	40
Fig. 5i	Normal	1.5	8192^2^	1	5.7	40
	Conductive	1.5	8192^2^	1	4	40
	Normal	1.4	4096^2^	1.6	7.5	40
	Conductive	1.4	4096^2^	2	6	40
	Normal	1.4	4096^2^	1	7.7	40
	Conductive	1.4	4096^2^	1	5.9	40
	Normal	1.4	4096^2^	1	8.7	60
	Conductive	1.4	4096^2^	1	7.4	60
	Normal	2	3072^2^	1	7.7	50
	Conductive	2	3072^2^	1	4.3	50
	Normal	2	3072^2^	1	4.8	50
	Conductive	2	3072^2^	1	4.3	50
